# The Efficacy of UV-C Disinfection in Decreasing Hospital-Acquired Infections and Bioburden in an Adult Burns Service

**DOI:** 10.3390/ebj7020025

**Published:** 2026-05-11

**Authors:** Elad Zvi, Melissa Neely, Louise Higgins, Maja Garcia, Melinda Pacquola, Eldho Paul, Alex Padiglione, Heather Cleland, Cheng Hean Lo

**Affiliations:** 1 Victorian Adult Burns Service, Alfred Health, 89 Commercial Road, Melbourne, VIC 3004, Australiacheng.lo@monash.edu (C.H.L.); 2 School of Public Health and Preventive Medicine, Monash University, Melbourne, VIC 3004, Australia; 3 Department of Surgery, Monash University, 99 Commercial Road, Melbourne, VIC 3004, Australia; 4 Department of Infectious Disease, Monash University, 99 Commercial Road, Melbourne, VIC 3004, Australia

**Keywords:** ultraviolet disinfection, hospital-acquired infection, burns unit, multidrug-resistant organisms, infection control

## Abstract

**Highlights:**

**What are the main findings?**

- Burn patients are highly vulnerable to hospital-acquired infections, and adjunctive UV-C room disinfection was associated with a lower rate of multidrug-resistant organism-related infections in an adult burns surgical ward.
- UV-C disinfection significantly reduced bacterial contamination on high-touch surfaces in patient rooms, including bed rails, door handles, sink faucets, and toilet seats.

**What are the implications of the main findings?**

- UV-C room disinfection may strengthen routine infection-prevention practice when used as an adjunct to standard hospital cleaning in adult burns services.
- These real-world findings support further controlled studies to determine the clinical value of UV-C disinfection in reducing infection risk among high-risk burn patients.

**Abstract:**

Background: Burn patients are highly susceptible to hospital-acquired infections (HAIs), and contaminated near-patient surfaces can act as reservoirs for multidrug-resistant organisms (MROs). Ultraviolet-C (UV-C) room disinfection is increasingly used as an adjunct to manual cleaning, but real-world data in adult burns settings remain limited. Methods: We evaluated adjunctive UV-C disinfection in a tertiary adult trauma and burns surgical ward using a two-part observational design. Part A compares MRO-related HAI incidence before UV-C implementation (12 May 2015–11 May 2020; retrospective) with its incidence after implementation (14 July 2020–13 July 2021; prospective). Part B is a matched pre/post environmental sampling study (December 2022–December 2024) of 44 vacant rooms. Paired swabs from a single randomised high-touch surface per room were collected immediately before and after UV-C disinfection and processed by an independent laboratory. Results: Part A included 7589 admissions (6415 before-UV-C; 1174 after-UV-C) with 2728 UV-C cycles delivered after implementation. MRO-related HAI incidence decreased from 18.3 to 10.2 per 1000 bed-days (*p* < 0.01). In Part B, the proportion of swabs with <10 CFU increased after UV-C disinfection (66% vs. 50%, *p* = 0.02). Among swabs with non-negligible baseline contamination and excluding increases, the median CFU reduction was 97% (SD 12%; *p* < 0.001), with no significant differences in reduction across sampled surface types. Conclusion: In an adult burns surgical ward, adjunctive UV-C disinfection was associated with reduced MRO-related HAI incidence and a substantial reduction in environmental bioburden on high-touch surfaces. These real-world findings support UV-C as a feasible adjunct to standard cleaning in high-risk burn services and inform future controlled evaluations.

## 1. Introduction

Surgical site infections (SSIs) are among the most common hospital-acquired infections (HAIs) globally, causing substantial morbidity and healthcare costs [1]. Infection prevention relies on surveillance and strategies to limit antimicrobial resistance and the spread of multidrug-resistant organisms (MROs), including prudent antimicrobial use, hand hygiene, aseptic techniques, reduced hospital stay, minimal use of invasive devices, adequate staffing, and active infection control programmes [2]. Burn patients are particularly susceptible to wound colonisation [3] by Gram-positive and Gram-negative organisms originating from endogenous enteric flora, exogenous contamination, or hospital-acquired sources [4], which can progress to local infection, increased tissue loss, graft loss, and systemic infection [5]. MROs pose an additional burden due to their ability to persist for prolonged periods in the hospital environment, especially on patient-room surfaces [6], underscoring the importance of environmental disinfection.

Alongside conventional cleaning, ultraviolet light technologies are increasingly used in healthcare settings. Ultraviolet-C (UV-C) is a widely accepted disinfection modality [7,8], delivering a measured UV dose that inactivates DNA and RNA via photon absorption and pyrimidine dimer formation from thymine and cytosine, resulting in microbial destruction [9,10,11]. While early UV-C disinfection (UV-C-D) studies demonstrated germicidal activity and reduced bioburden, more recent findings have been mixed [7,10,12]. According to a recent meta-analysis, some studies showed that UV-C-D can be effective at reducing Gram-negative rod infections specifically [6] but did not find any evidence of benefit in using UV-C disinfectant systems in health care facilities to reduce HAIs, or any significant benefit when using UV-C-D as an adjunct to standard hospital cleaning protocols. Further studies have investigated the implementation of UV-C-D, including comparisons of disinfection levels [13] and the amount of UV-C light needed [14]. Few studies directly measure the impact of UV-C-D on HAI rates [15]. Furthermore, only one study to date has investigated the role of UV-C-D disinfection in the environment of patients with burns, which measured the ICU setting [16].

The aim of this study was to report our experience with UV-C-D in a tertiary adult burn centre in Melbourne, Australia, conducted before, during and after the COVID-19 pandemic.

## 2. Materials and Methods

### 2.1. Study Design

Part A of the study compared data (HAIs involving MROs) before UV-C-D implementation (collected retrospectively) with data after UV-C implementation (collected prospectively). Part B was a non-randomised, matched, prospective study, investigating changes to bacterial counts and organisms swabbed from patient room surfaces before and after UV-C-D implementation. Part A and Part B represent complementary components of a single study.

Evaluating UV-C implementation in our burns ward. Part A evaluates the association of UV-C cleaning with clinical infection outcomes, while Part B assesses the environmental bioburden effect that may plausibly underlie those clinical findings.

Data was collected immediately before and after UV-C cleaning. The planned sample size for Part B of the study was a minimum of 40 patient rooms. Rooms were eligible if they had been occupied by a patient with confirmed colonisation, defined as a routine clinical culture positive for an organism, and if the room was available for environmental sampling while vacant, either during temporary patient transfer to theatre or between patient occupations. Eligible rooms were sampled consecutively, where feasible, during the study period until the target sample size was reached. This sample size provides 80% power to detect a difference equivalent to 0.45 standard deviation units on a continuous outcome between cleaning periods with a 2-sided *p*-value of 0.05. Given the exploratory nature of this study, the proposed sample size was deemed sufficient to provide some preliminary data on intervention efficacy.

### 2.2. Setting

This study was conducted in a single ward (Ward 6 West) at The Alfred Hospital, a tertiary adult trauma and burn centre in Melbourne, Australia. This ward has a total of 29 beds in single and multiple rooms and incorporates the Victorian Adult Burns Service (VABS), and other specialities including plastic and reconstructive surgery, vascular surgery, ear, nose and throat surgery, urology, and general surgery. VABS is the state-wide adult specialist burns service in Melbourne, Australia, admitting approximately 350 patients with acute burns annually.

Data from Part A was collected from 2015 to 2021. This included a five-year baseline, or pre-implementation, period (12 May 2015–11 May 2020) and a one-year intervention, or post-implementation, period (14 July 2020–13 July 2021). The study excluded a two-month wash-in period (the first treatment cycle took place on 12 May 2020, whilst formal ward support staff training took place on 13 July 2020) during which UV-C technology was being introduced [17]. Part B of the study was conducted from December 2022 until December 2024.

### 2.3. UV-C Disinfection

The Surfacide Helios^®^ UV-C-D system is a multiple emitter, automated, and remotely operated system that self-adjusts to the size and content of the room and delivers appropriate doses of UV-C energy.

The Surfacide Helios^®^ UV-C-D system, manufactured by Surfacide LLC. (Waukesha, WI, USA), is a multi-emitter, automated, and remotely operated platform designed for whole-room disinfection. The system consists of three mobile UV-C towers that are positioned around the patient. Using a laser, the device maps the room geometry, detects fixed objects, and automatically adjusts treatment parameters, including cycle duration and UV-C dose, to account for room size and contents. This allows for standardised and reproducible delivery of germicidal UV-C energy to high-touch surfaces and environmental reservoirs while minimising operator variability and ensuring safety during operation. In our setting, UV-C operating time was 10 min.

At The Alfred Hospital, UV-C-D was implemented as an adjunct, following standard hospital cleaning protocols. Physical cleaning includes manual removal of macroscopic or visible contamination, and the use of disinfection agents, including alcohol wipes with 70% isopropyl alcohol, Clinell^®^ Sporicidal and Clinell^®^ Universal wipes (GAMA Healthcare Australia, Notting Hill, VIC, Australia), Viraclean^®^ Disinfectant (benzalkonium chloride 4.255 g/L) (Whiteley, North Sydney, NSW, Australia) and Actichlor™ Plus Disinfectant Solution (EcoLab Healthcare, Cheshire, UK). All non-dedicated equipment and accessories that come into contact with a patient or a patient’s environment are cleaned and disinfected. Cleaning of patient rooms and high-touch surfaces takes place daily and upon patient transfer or discharge. Shared patient equipment and items are cleaned before removal from the room between patient uses. Appropriate personal protective equipment is worn by staff undertaking cleaning or disinfection of the environment or equipment. Although the COVID-19 pandemic triggered many infection prevention and treatment changes, the stewardship protocols remained without change.

### 2.4. Inclusion Criteria

In Part A, patients were included if they were admitted to Ward 6 West during the 5-year pre-implementation period and the subsequent post-implementation period. For Part B, patients’ rooms were included in the study if (1) the patient had a confirmed colonisation (culture positive for organism in order to allow exploration of a possible correlation between patient colonisation and contamination of the immediate room environment) and (2) the room was vacant whilst the patient was temporarily transferred to the operating theatre, or room was being cleaned between patient occupations.

### 2.5. Primary Endpoints

For Part A of the study, the primary endpoint was the number of HAIs involving MROs, including *Acinetobacter baumannii*, *Klebsiella pneumoniae*, methicillin-resistant *Staphylococcus aureus* (MRSA), vancomycin-resistant *Enterococcus* (VRE), *Pseudomonas aeruginosa*, *Stenotrophomonas*, Extended spectrum beta lactamase (ESBL), *Clostridioides difficile*, *Enterobacter*, *Citrobacter*, *Serratia* and *E. coli.* Results are expressed as the number of HAIs, rate of HAIs (cases per 1000 patient-days; divide the total number of HAIs by the total number of patient days and multiply by 1000). For Part B, the primary endpoint was the bacterial count in environmental samples (patient room surfaces) taken before and after UV-C-D. This is expressed as % positive cultures, colony-forming units (CFUs), and % decrease in CFUs.

### 2.6. Data Collection

For Part A, data were extracted from the electronic medical record for patients who met eligibility criteria between 2015 and 2021. Data collected include patient demographics (age, gender, admission details), average hospital length of stay (per month), and patient days or occupied bed days (per month). In Part B of the study, 44 vacant rooms undergoing standard hospital cleaning followed by UV-C-D were identified. A set of two swab samples was taken from each room, (1) before UV-C-D and (2) after UV-C-D, from high-touch surfaces (randomised to bed rail, sink faucet, toilet seat, door handle). Each set of swab samples was taken from the same high-touch surface in each room, and cleaning personnel were blinded as to which high-touch surface was swabbed. The swab samples were sent to an external independent laboratory, ALS Global (Scoresby, Victoria, Australia), for testing. Laboratory personnel were blinded as to whether samples were taken before or after UV-C-D.

### 2.7. Data Analysis

All analyses were performed using SAS software version 9.4 (SAS Institute, Cary, NC, USA). Comparisons between groups (pre- and post-) were made using Student’s t-test or Wilcoxon rank-sum test as appropriate for continuous variables and chi-square or Fisher’s exact test for categorical variables. Incidence rates were compared using Poisson regression.

Comparisons between before and after cleaning periods were made using the Wilcoxon signed rank test for continuous variables and McNemar’s test for categorical variables. To compare rates of decrease between types of surfaces swabbed, one-way ANOVA was used. No adjustment was made for multiple outcome comparisons. A two-sided *p*-value of 0.05 was chosen to indicate statistical significance.

Environmental swabs were processed by an external independent NATA-accredited laboratory (ALS Global, Scoresby, VIC, Australia). Total aerobic microbial count was determined using the laboratory’s validated TAMC method (QWI-PM0003), based on pour plate and filtration techniques. Samples underwent serial dilution with 1 mL aliquots inoculated onto Petri dishes, overlaid with Tryptone Soya Agar, and incubated aerobically at 30–35 °C for 5 days; where filtration was used, the filtered membrane was placed onto TSA and incubated under the same conditions. Results were reported as CFUs per amount tested, with a reporting threshold of <10. Microbial identification of recovered isolates was performed using MALDI-TOF Biotyper (QWI-FM0135) (Bruker Daltonik GmbH & Co. KG, Bremen, Germany).

## 3. Results

### 3.1. Part A: Incidence of HAIs Before and After UV-C-D

A total of 7589 patients were included in the analysis, with 6415 patients included before UV-C-D implementation and 1174 patients after UV-C-D implementation (Table 1). During the UV-C-D implementation period, 2728 UV-C-D cycles were completed. There were a total of 1005 HAIs involving MROs (909 HAIs before the implementation period, 96 after the UV-C-D implementation period) (Table 2). *Pseudomonas aeruginosa* (27.8%) was the most common infection before the UV-C-D implementation period, whereas *Enterobacter* species (26%) was the most common causative organism after the UV-C-D implementation period. Incidence of HAIs involving MROs calculated per 1000 bed days was 18.3 before the UV-C-D implementation period and 10.2 after the UV-C-D implementation period (*p* < 0.01).

### 3.2. Part B: Characterising and Comparing Microbials on Patients and Their Environment

Forty-four sets of samples were collected from patients’ environments (Table 3). Before UV-C-D, the total microbial count of the positive 22 swabs (with 10 or more CFUs) had a mean of 634.71 CFUs (range 10–4000 CFUs). The remaining 22 negative swabs returned microbial counts of <10 CFUs. After UV-C-D, the mean total microbial count across 15 swabs (with 10 or more CFUs) was 77.7 CFUs (range 10–220 CFUs).

Nineteen out of 44 sets of swabs revealed a decrease in bacterial CFUs before and after UV-C-D (*p* < 0.001). Of the remaining 25 sets of swabs, 2 revealed no change in CFUs, 21 had negligible bacterial counts (<10) before and after UV-C-D, whilst two swabs showed an increase in CFUs. There were significantly more swabs after UV-C-D with <10 CFUs compared to before UV-C-D (66% vs. 50%, *p* = 0.02). When swabs that showed negligible (<10) bacterial counts before and after UV-C-D and two swabs that showed an increase in bacterial CFUs were further excluded (to better evaluate the decrease rate), the median rate of decrease before and after UV-C-D was 97% (SD 12%).

### 3.3. UV-C-D Rates Across Different Surfaces

We compared the rate of decrease across different surfaces in hospital rooms that were UV-C-D cleaned and swabbed for this study. Sites swabbed in patient environments included door handles (28%), cupboard surfaces (24%), sink faucets (24%) and toilet seats (24%). Out of 44 swabs, seven swabs did not have information regarding surface type. These swabs were excluded, and a comparison was performed of the bacterial count in the remaining 37 sets of swabs with 10 or more CFUs before UV-C-D, and a decrease in bacterial count before and after disinfection (excluding the single swab with an increase in CFUs). There were no significant differences in the rates of decrease in bacterial CFUs across surfaces, even following the exclusion of swabs that showed negligible counts before UV-C-D (<10 CFUs) and the exclusion of one outlier set of swabs with an increase in bacterial count (Table 4).

Nineteen microorganisms were identified in patients included in the study, with *Staphylococcus aureus* being the most common (*n* = 20, 45%), followed by *Klebsiella pneumonia* (*n* = 7, 16%) (Table 5). Before UV-C-D, 12 different microorganisms were identified in surfaces swabbed, with *Staphylococcus epidermidis* being the most frequently identified microorganism (*n* = 7, 16%). We compared the microbes identified on patients and those identified on patient room surfaces before UV-C-D and found that the same organism was identified in both samples on one occasion only (*Staphylococcus aureus*). After UV-C-D, 12 different microorganisms were also identified in the patient environment. Nine organisms identified before UV-C-D were undetectable after UV-C-D. *Staphylococcus epidermidis* was the most frequently seen microorganism after UV-C-D, albeit in fewer swabs compared to before UV-C-D (*n* = 3, 7%). There was no significant difference in the frequency of microorganisms detected before and after UV-C-D (*p* = 0.423). There were nine microorganisms that were only identified after UV-C-D.

## 4. Discussion

This study provides important insights into the implementation of UV-C-D in a tertiary adult burn and trauma surgical ward, addressing a patient population at high risk of infection due to wound colonisation, immune dysfunction, prolonged hospitalisation, and repeated surgical interventions. As environmental surfaces serve as persistent reservoirs for pathogenic and multidrug-resistant organisms, strategies that reduce environmental bioburden may represent a clinically meaningful adjunct to infection prevention efforts in burn units.

In Part A of the study, before-UV-C-D-implementation data included patients prior to and following the onset of the COVID-19 global pandemic. The COVID-19 pandemic led to a significant review of infection prevention strategies and increased implementation of preventive measures and contact precautions in hospitals globally. The impact of the COVID-19 pandemic and ensuing infection prevention strategies on HAIs is complex. Data from the United States showed that there was an increase in HAIs during the pandemic due to the increased numbers of hospitalisations and a more varied demographic of patients being hospitalised [18]. A study based in Victoria, Australia, found a decrease in specific infections pre- and post-COVID (*S. aureus* bacteraemias and *C. diff* infections) but found no significant change in surgical site and line-associated infections [19]. Another study based in a metropolitan hospital in Victoria, Australia, found no change in HAI incidence following the introduction of infection prevention measures [20]. Ongoing infection prevention measures, such as contact precautions implemented at our hospital following the pandemic, may have contributed to the decrease in HAIs after UV-C-D implementation, but their impact on HAI rates may be challenging to measure. Nevertheless, our study demonstrated a reduction in HAI incidence following the implementation of UV-C-D. This finding is consistent with previous studies, indicating that UV-C-D can be associated with a reduction in HAIs [16].

Part B of our study demonstrated a decrease in bacterial environmental colonisation after UV-C-D. Prior studies have demonstrated a similar impact but in a variety of settings and patient populations [21]. To date, our study is the first to explore the quantitative impact of UV-C-D on environmental colonisation in the rooms of patients with burns. Our data demonstrated no significant differences in the magnitude of CFU reduction following UV-C exposure across different surface types, suggesting that UV-C-D may be equally effective across a range of surfaces and clinical areas. All four surfaces exhibited susceptibility to UV-C-D.

There was a discrepancy in the organisms identified in patients included in study Part B and room surface swabs taken before UV-C-D (Table 5). Similar discordance has been previously described in the literature. Environmental contamination from colonised wounds is highest during dressing changes and much lower at other times, meaning that point-prevalence sampling may miss transiently shed organisms [22]. Survival on dry inanimate surfaces is highly organism- and surface-dependent; some wound pathogens have reduced persistence on dry high-touch surfaces and are therefore less likely to be cultured from the environment [23]. Studies of MRSA-colonised patients also demonstrated only partial overlap between strains isolated from patients and those recovered from their immediate environment, indicating that environmental sampling underestimates the true burden of patient-associated contamination [24,25]. Furthermore, the sensitivity of surface-sampling techniques is limited and strongly influenced by the sampling device, area and culture methods used [26]. In our study, patients may have developed their HAI from other parts of the hospital rather than their occupied room. Despite blinding of cleaning personnel, manual cleaning practices may have improved following the implementation of the study. The patient room may have been treated recently with UV-C-D prior to the swabs being taken. Due to the small number of organisms isolated, it is difficult to form any definitive conclusions in this regard. However, some of these scenarios demonstrate the importance of implementing UV-C-D as an adjunct to standard hospital cleaning protocols.

In our study, the shift in organism profile observed after UV-C-D is consistent with published data showing that UV disinfection can selectively inactivate more susceptible taxa, thereby altering microbial community composition [27,28]. Differential UV-C susceptibility between species, [29,30] heterogeneity in delivered dose due to shadowing and surface topography [14,31], protection of biofilm-associated organisms [32], and subsequent re-colonisation from patients, staff and airflows with a different baseline flora [16] are all mechanisms supported by previous work that may together explain the changed organism distribution we observed before and after UV-C-D.

The exploratory nature of this study has limitations. In Part A, the pre-implementation data were collected over a longer period of time (5 years) compared to post-implementation (1 year). Although Incidence was expressed as HAIs per 1000 patient-days, there is a possibility that this disparity in data collection periods may have introduced bias from year-to-year fluctuations in HAIs that were not adequately captured in the year following the collection of UV-C implementation data. In addition, Part A was conducted in a mixed surgical ward rather than a dedicated burns-only ward, with burn patients comprising approximately 30% of the population, plastic surgery 24%, and the remainder from vascular surgery, ear, nose and throat surgery, urology, and general surgery. Different surgical subspecialities may have different colonisation profiles and infection risks. Part B of the study was limited by a small sample size; however, it was sufficient to provide preliminary data on intervention efficacy. A direct comparison of UV-C-D against standard hospital cleaning alone would be interesting and informative.

Further studies to prove and quantify a direct causative relationship between UV-C cleaning and HAI, or between patient room contamination and HAI, would be relevant. While the observational design, single-centre setting and relatively small environmental sample mean that our findings should be interpreted with some caution, they reflect real-world practice during a period of evolving infection prevention measures.

## 5. Conclusions

In our centre, the introduction of adjunctive UV-C room disinfection was associated with lower rates of MRO-related HAIs and a reduction in environmental bacterial bioburden, with similar effects across several high-touch surfaces. Overall, our results support UV-C-D as a feasible adjunct to standard cleaning in burns services and provide a basis for larger, controlled studies to further define its role in infection prevention.

## Figures and Tables

**Table 1:** Demographic data for patients included in Part A.

	Before-Implementation	After-Implementation
Total number of patients (N)	6415	1147
Age at admission (mean, range)	52.7 (15–103)	50.9 (16–101)
Female (N, %)	2115 (33)	360 (30.7)
Length of stay in days (mean, SD)	8 (14)	8 (13)

**Table 2:** Summary of HAIs involving MROs.

	HAIs Involving MROs (N = 1005)		
	Before UV-C-D	After UV-C-D	*p*
MRO-incidence per 1000 bed days	18.3	10.2	<0.01
MRO identified in HAIs N (%)	909 (90.4)	96 (9.6)	
*Acinetobacter baumanii*	64 (7.0)	8 (8.3)	
*Citrobacter* species	23 (2.5)	1 (1.0)	
*Clostridioides difficile*	33 (3.6)	5 (5.2)	
*Enterobacter* species	127 (14.0)	25 (26.0)	
*ESBL-producing bacteria*	41 (4.5)	8 (8.3)	
*Escherichia coli*	118 (13.0)	10 (10.4)	
*Klebsiella pneumoniae*	64 (7.0)	8 (8.3)	
MRSA	60 (6.6)	10 (10.4)	
*Pseudomonas aeruginosa*	253 (27.8)	2 (2.1)	
*Serratia* species	37 (4.1)	3 (3.1)	
*Stenotrophomonas* species	50 (5.5)	12 (12.5)	
VRE	39 (4.3)	4 (4.2)	

**Table 3:** Total microbial counts from 44 sets of samples taken before and after UV-C disinfection.

Total Microbial Count	Before UV-C-D (CFUs/Swab, %)	After UV-C-D (CFUs/Swab, %)	
10 or more	22 (50%)	15 (34%)	
<10	22 (50%)	29 (66%)	*p* = 0.02

**Table 4:** Rate of decrease in bacterial count before and after UV-C-D across different room surfaces.

Surface Swabbed	Mean Rate of Decrease % (SD)	N = 37
Cupboard surface	21 (41)	9
Door handle	36 (47)	10
Sink faucet/tap/handle	61 (46)	9
Toilet seat	29 (45)	9
	*p* = 0.28	
Following exclusion of swabs with <10 CFUs before UV-C-D		N = 16
Cupboard surface	93 (10)	2
Door handle	91 (9)	4
Sink faucet/tap/handle	78 (36)	7
Toilet seat	86 (23)	3
	*p* = 0.85	
Following exclusion of swabs with <10 CFUs before UV-C-D and swabs that showed an increase		N = 15
Cupboard surface	93 (10)	2
Door handle	91 (9)	4
Sink faucet/tap/handle	91 (13)	6
Toilet seat	86 (23)	3
	*p* = 0.96	

**Table 5:** Microorganisms identified in 44 patients and room surfaces swabbed in Part B of the study.

Microorganism Identified	Organisms Identified in Patients Included in Study, N (%)	Before UV-C-D Swab N (%)	After UV-C-D Swab N (%)
*Acinetobacter*	2 (5)	0	0
*Alkalihalobacillus gibsonii*	0	0	1 (2)
*Bacillus cereus*	0	1 (2)	1 (2)
*Bacillus subtilis*	0	1 (2)	0
*Brachybacterium muris*	0	0	1 (2)
*Brevundimonas nasdae*	0	1 (2)	0
*Candida albican*	1 (2)	0	0
*Citrobacter freundii*	1 (2)	0	0
*E. coli*	2 (5)	0	0
*Enterobacter cloacae*	3 (7)	0	0
*Enterococcus faecium*	0	0	1 (2)
*Klebsiella oxytoca*	2 (5)	0	0
*Klebsiella pneumonia*	7 (16)	0	0
*Kocuria rhizophila*	0	0	1 (2)
*Lacticaseibacillus paracasei*	0	0	1 (2)
*Lactobacillus rhamnosus*	0	0	1 (2)
*Micrococcus flavus*	0	0	1 (2)
*Micrococcus luteus*	0	1 (2)	0
*Micrococcus terreus*	0	1 (2)	1 (2)
*Moraxella osloensis*-	0	0	1 (2)
*Musicillium theobromae*	0	0	1 (2)
*Ochrobactrum tritici*	0	1 (2)	0
*Penicillium steckii*	0	1 (2)	0
*Pseudomonas alcaligenes*	1 (2)	0	0
*Pseudomonas* species	1 (2)	0	0
*Pseudomonas stutzeri*	1 (2)	0	0
*Pseudomonas aeruginosa*	5 (11)	0	0
*Serratia marcescens*	1 (2)	0	0
*Staph lugdunensis*	1 (2)	0	0
*Staphylococcus aureus*	20 (45)	1 (2)	0
*Staphylococcus capitis*	0	4 (9)	0
*Staphylococcus epidermidis*	0	7 (16)	3 (7)
*Staphylococcus haemolyticus*	0	2 (5)	0
*Staphylococcus hominis*	0	1 (2)	0
*Stenotrophomonas*	2 (5)	0	0
Strep A (Group A streptococcus)	2 (5)	0	0
*Strep agalatiae*	1 (2)	0	0
*Strep dysgalactiae*	1 (2)	0	0
Yeast species	1 (2)	0	0

## Data Availability

The data presented in this study are available on reasonable request from the corresponding author. The data are not publicly available due to privacy and ethical restrictions.

## Abbreviations

The following abbreviations are used in this manuscript:

UV-C	Ultraviolet-C
UV-C-D	Ultraviolet-C Disinfection
HAI/HAIs	Hospital-Acquired Infection(s)
MRO/MROs	Multidrug-Resistant Organism(s)
CFU	Colony Forming Units
MRSA	Methicillin-Resistant Staphylococcus aureus
VRE	Vancomycin-Resistant Enterococcus
ESBL	Extended-Spectrum Beta-Lactamase
ICU	Intensive Care Unit
VABS	Victorian Adult Burns Service
SD	Standard Deviation
ANOVA	Analysis of Variance
